# High monetary reward rates and caloric rewards decrease temporal persistence

**DOI:** 10.1098/rspb.2016.2759

**Published:** 2017-02-22

**Authors:** Bowen J. Fung, Stefan Bode, Carsten Murawski

**Affiliations:** 1 Melbourne School of Psychological Sciences, The University of Melbourne, Melbourne, Victoria 3010, Australia; 2 Department of Finance, The University of Melbourne, Melbourne, Victoria 3010, Australia

**Keywords:** impulsivity, foraging, opportunity cost, reward, energy budget rule, interval timing

## Abstract

Temporal persistence refers to an individual's capacity to wait for future rewards, while forgoing possible alternatives. This requires a trade-off between the potential value of delayed rewards and opportunity costs, and is relevant to many real-world decisions, such as dieting. Theoretical models have previously suggested that high monetary reward rates, or positive energy balance, may result in decreased temporal persistence. In our study, 50 fasted participants engaged in a temporal persistence task, incentivised with monetary rewards. In alternating blocks of this task, rewards were delivered at delays drawn randomly from distributions with either a lower or higher maximum reward rate. During some blocks participants received either a caloric drink or water. We used survival analysis to estimate participants' probability of quitting conditional on the delay distribution and the consumed liquid. Participants had a higher probability of quitting in blocks with the higher reward rate. Furthermore, participants who consumed the caloric drink had a higher probability of quitting than those who consumed water. Our results support the predictions from the theoretical models, and importantly, suggest that both higher monetary reward rates and physiologically relevant rewards can decrease temporal persistence, which is a crucial determinant for survival in many species.

## 1. Introduction

Patience is often treated as a virtue, as acting patiently can lead to long-term gains. Conversely, acting impatiently can also be advantageous in some circumstances, particularly when the potential time spent waiting for rewards is uncertain, or when the value of alternative behaviours is high. Temporal persistence refers to the duration of time individuals will wait in the face of increasing opportunity costs, and is a crucial aspect of many real-world decisions. For example, an individual may decide to wait for a bus, but after waiting for 15 min, may abandon waiting and take a taxi instead, despite the fact that this option was available from the beginning. One explanation for this apparent inconsistency is that real-world delays carry implicit uncertainty [1,2], and that individuals' beliefs about potential waiting times are continuously updated by experience of the environment [3–5]. From the perspective of optimal foraging—where animals try to maximize their rate of reward intake while minimizing the opportunity cost of time [6]—any decision to abandon waiting should occur once the opportunity cost of time outweighs the potential future reward [7–9]. In our example, this might mean giving up waiting for the bus after the opportunity cost of waiting exceeds the cost of a taxi.

Not much is known about how humans calculate temporal opportunity costs. In one neurocomputational model, opportunity cost is directly proportional to the recently experienced average reward rate [10]. This is because higher experienced reward rates signal high reward availability, thus periods of inactivity are more costly, relative to when reward availability is lower. Under this framework, actions should be performed more quickly (i.e. with more vigour) in order to offset the relatively high temporal opportunity cost [11]. The model proposes that this increase in speed is facilitated by an increase in tonic dopamine in the striatum [12,13], and therefore tonic dopamine levels are proportional to both experienced reward rate and subjective temporal opportunity costs. Previous imaging studies have provided support for this idea by showing covariation between average reward rate and the tonic activity of dopaminergic midbrain areas [12,14]. This encoding of opportunity cost by tonic dopamine is consistent with the reported effects of dopaminergic manipulations on perceived durations: dopamine agonists cause overestimations of time, which ought to increase subjective opportunity costs, while dopamine antagonists cause underestimations of time, which ought to have the opposite effect [15,16]. The effect of dopaminergic agents on perceived durations and subjective opportunity costs may also help to explain findings in delay discounting, where enhancing dopamine levels can lead to more impulsive choices [17]. Thus, the putative dopaminergic encoding of opportunity cost could be viewed as equivalent to an urgency signal [18], one which affects measurements across multiple timing domains, including reaction times [19], duration perception [15,16,19], delay discounting [17] and possibly temporal persistence.

Another framework, risk-sensitive foraging theory [20], also outlines how temporal persistence might change in response to internal signals, such as an increase or decrease in energy balance [21]. According to this framework (widely referred to as the energy budget rule), organisms with a negative energy balance should take more foraging risks (be risk-prone) in order to minimize the probability of starvation, while organisms with a positive energy balance should be risk-averse [22]. In humans, this has been supported by evidence showing that a positive energy balance increases risk-aversion for the acquisition of both monetary [23] and physiological rewards [24]. Any potential delay to reward carries implicit risk, and it has been proposed that risk and delay might be psychologically analogous, if not equivalent [1,2]. Thus, under the energy budget rule, it follows that organisms with more energy should exhibit aversion to delay, and decreased temporal persistence, relative to organisms with less energy. However, to our knowledge, whether energy balance affects temporal persistence in humans has not been tested.

The two frameworks discussed above make testable predictions. Firstly, temporal persistence should decrease during periods in which experienced average reward rates are high, due to increased subjective opportunity costs. Secondly, temporal persistence should also decrease when energy balance is high, due to an increase in aversion to delay. In this study, we tested these theoretical predictions independently, using a modified version of a temporal persistence task [3]. Prior work based on this task has shown that individuals adapt their temporal persistence to their experience with delays drawn from different distributions [3]. In our experiment, we used different distributions of delays to manipulate the reward rate between blocks. Additionally, in between blocks our fasted participants received either a sweet, caloric solution or water as a control, in order to manipulate relative energy balance. These two manipulations allowed us to determine whether high average reward rates or the consumption of a caloric solution would decrease temporal persistence.

## 2. Material and methods

### (a) Participants

Fifty participants from the general population (mean age 23, range 18–40, 30 female, five left-handed) were recruited via advertisement at The University of Melbourne. All participants reported no dietary restrictions (fructose intolerance, diabetes, fluid imbalance or phenylketonuria) and provided written informed consent. Participants were instructed to refrain from eating or drinking for 4 h prior to the experiment in order to decrease satiety. Participants were compensated with at least AUD 10 for their participation, as well as an additional amount contingent on their task performance (maximum total AUD 20). The study protocol was approved by The University of Melbourne Human Research Ethics Committee (no. 1441974).

### (b) Stimuli, apparatus and procedure

**Figure 1.**
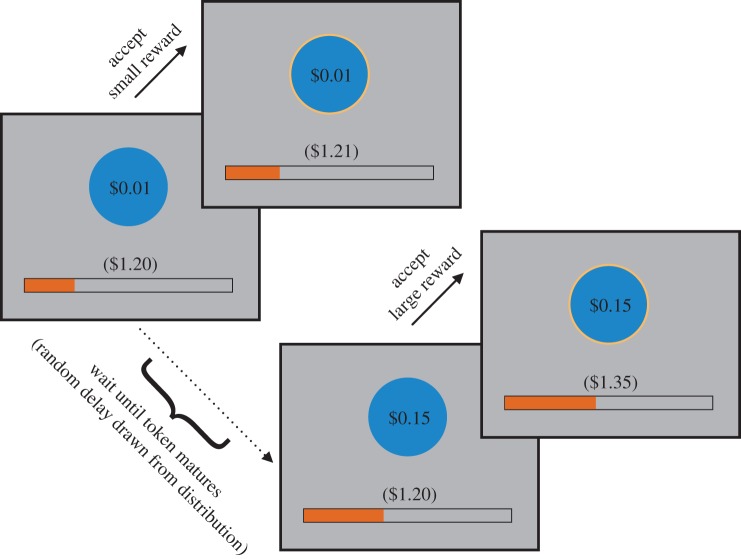
Temporal persistence task paradigm. In each block of the task, tokens were serially presented to participants. Initially, these tokens were valued at $0.01. At any time, participants could accept this token and receive the small reward. Alternatively, the participant could wait for a random delay until the token matured to a value of $0.15, and then accept this larger reward. Once a token was accepted, there was an inter-trial interval of 2 s before the presentation of the next token. The total reward earned and the cumulative duration of the block were displayed below the token.

We measured temporal persistence using a task in which participants could wait for a randomly timed, delayed, monetary reward ($0.15) or could at any time decide to ‘quit’ waiting and accept a small, immediate, monetary reward ($0.01; figure 1; [3,7]). In each block of this task, participants were given a fixed amount of time (5 min) to earn these rewards. A bar was displayed at the bottom of the screen indicating the cumulative duration of the block. At 5 min, the bar filled and the block was terminated. During these 5 min, tokens would appear on the screen serially, one at a time. Initially, they would be valued at $0.01, but after an uncertain delay (see below for procedure) would mature to a value of $0.15. At any point, participants could press a button to take the token, and earn the corresponding value. After a 2 s inter-trial interval, a new token would appear. Participants were instructed to maximize their earnings during each 5-min block using whatever strategy they preferred. For example, a participant may have adopted a policy to continuously accept the small tokens, which would allow them to earn $0.01 every 2 s. Alternatively, by always waiting until the tokens matured before taking them, more would be earned with each token, but this process would have a high opportunity cost (the potential reward forgone by waiting). Participants were told that they would be paid exactly half of their total winnings in the task, but guaranteed a minimum payment of AUD 10.

In each block, we determined the timing of the delayed reward by sampling from one of two different delay distributions, which alternated between blocks. These two different distributions constituted two different timing environments in which the optimal quitting time and potential reward rate differed (optimal quitting times and average earnings under this policy were derived by performing a normative analysis as detailed in [3]). The possible delays for the first timing environment were described by a uniform distribution on the interval 0 to 12 s. The optimal policy in this environment was to always wait for the full 12 s, which would have earned approximately $5.58 in each block. The delay distribution of the second, ‘heavy-tailed’ timing environment was described by a generalized Pareto distribution of the form:2.1
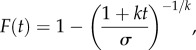
with parameters *k* = 8, *σ* = 3.4 and the upper bound set at 90 s. The optimal quitting policy in this environment was to quit at 2.13 s, which would have earned approximately $5.70 in each block. Thus, the potential reward rate of the heavy-tailed timing environment was higher than in the uniform environment. To ensure full exposure to the range of each distribution, delays were not sampled entirely randomly, but were instead sampled randomly from each quartile of the distribution in a pseudo-random order [3]. The different timing environments were represented by different coloured tokens (either orange or blue), and while participants were not explicitly told about the different delay distributions, they were instructed that their strategy may have to change depending on the colour of the token. Upon debriefing, participants reported using different strategies for each timing environment, commensurate with our analyses below.

Participants first completed two practice blocks of the task (one block of each timing environment), prior to completing a further eight blocks (10 blocks in total). In between blocks 5 through 8, participants consumed either 75 ml of water (control condition) or 75 ml of a sweet, caloric solution (caloric reward condition). This solution consisted of 6.4 g of a maltodextrin (a tasteless carbohydrate) and 20 mg of aspartame per 100 ml water. This solution was intended to mimic ecologically feasible rewards in terms of both energy content and sweetness, while allowing for future studies which could disassociate these two aspects. It was made clear to the participants that they could refrain from the experiment if they found the drink unpalatable. No participants were excluded on this basis, and 18 of the 25 participants in the caloric condition reported that they found the caloric drink ‘rewarding’, as opposed to ‘not rewarding’, upon debriefing. Importantly, in order to minimize any possible confounds between drink consumption and time-on-task, blocks in which participants did not consume liquid were balanced before and after blocks in which participants did consume liquid. After completion of the experimental task, participants were debriefed, and their total winnings computed and paid.

The Psychophysics Toolbox [25] running on MATLAB v. 8.4 was used for stimulus presentation.

### (c) Data analysis

As quitting decisions were the data of interest, we excluded data from two participants (both in the caloric reward condition) who failed to make any quitting decisions in either distribution, during the drinking blocks. The final sample included data from 48 participants.

While instances where participants abandoned waiting provided a direct measure of their persistence (or lack thereof), the exclusive use of these events neglects the information in instances where the token matured prior to a quitting decision. These events indicate that participants were willing to wait at least as long as it took for the large monetary reward to become available, and are equivalent to censored data in survival analysis. Thus, we adopted a technique from survival analysis, known as frailty modelling [26], which allowed us to model the probability of quitting as a function of time, while accounting for censored data, as well as accounting for dependence of events within subjects (the frailty is comparable with a random effect). Specifically, we used a semiparametric penalized likelihood estimation with a lognormal frailty as we assumed that our random effects were normally distributed. The hazard function for this shared frailty model was2.2
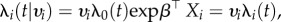
where λ_0_(*t*) is the baseline hazard function, *X_i_* the covariate vector associated with the vector of regression parameters *β*^⊤^ and *υ_i_* is the random effect associated with the *i*-th individual.

We first assessed the effect of timing environment (monetary reward rate) on temporal persistence, using only data from blocks in which participants did not drink, by including timing environment as a regressor in the frailty model. To assess whether caloric reward altered temporal persistence, we created an additional model using only the data from the blocks in which participants consumed liquid, and two further models to calculate hazard ratios for each timing environment separately. In an additional analysis, we also created separate models for each timing environment and each liquid type, in order to assess whether consumption of liquid (relative to not consuming liquid) had an effect on temporal persistence within each treatment group. Hazard ratios were calculated from the coefficients in all of the above models, and reported along with 95% CIs. We also performed a control analysis to determine whether the differences in the distribution of rewards in each timing environment affected the actual rate of reward experienced by participants, and whether either treatment affected reward rates within each timing environment. To calculate average experienced reward rate, we divided the cumulative reward by the cumulative duration at each decision point, and averaged these values over each block.

All statistical analyses were performed using R (v. 3.2.1). All frailty models were created using the statistical package frailtypack [27], with number of knots set to the default of 7, and the smoothing parameter estimated by cross-validation. For all statistical tests, the significance level was set to *p* < 0.05, and multiple comparisons were corrected using the Holm–Bonferroni method [28].

## 3. Results

### (a) Differences between timing environments

Firstly, we determined whether there were differences in behaviour between the two timing environments. To this end, we estimated a shared lognormal frailty model with timing environment as a regressor, using data pooled from both treatment conditions, and only from the blocks in which participants did not consume liquid.

**Figure 2.**
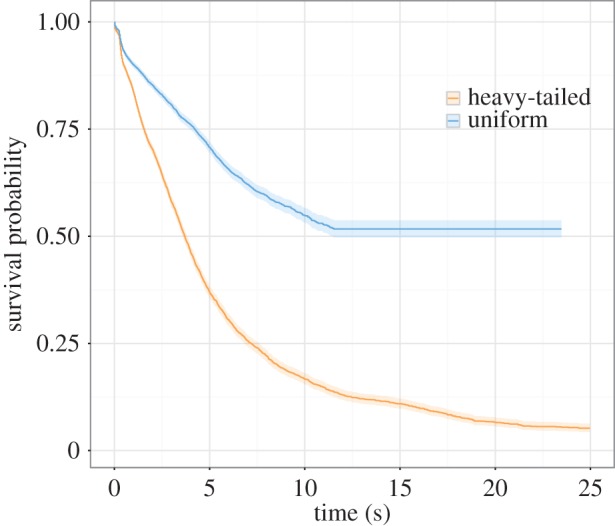
Survivor functions for each timing environment. The probability of waiting is plotted as a function of time for the uniform and heavy-tailed timing environments. The probability of waiting was lower in the heavy-tailed timing environment relative to the uniform timing environment. Note that functions shaded confidence intervals are estimated from pooled data.

The model revealed a significant effect of the heavy-tailed timing environment (*β* = 1.11, s.e. = 0.03, *z* = 39.95, *p* < 0.001). The hazard ratio was 3.05 (CI = 2.89–3.22), indicating that the probability of quitting was around three times as high in the heavy-tailed timing environment compared to the uniform timing environment. This suggests that individuals decreased their temporal persistence when reward rates were higher. Survivor functions estimated using pooled data for each timing environment are shown in figure 2.

We then performed a control analysis to confirm that the differences between the timing environments affected the actual rate of reward experienced by participants. As we were interested in the effect of recently experienced reward rates on each decision, we calculated reward rate by dividing the cumulative reward by the cumulative duration at each decision point. We compared the mean reward rates between timing environments using data pooled from both treatment conditions, and only from the blocks in which participants did not consume liquid.

The mean experienced reward rate in the uniform timing environment was 1.52 cents s^−1^ (s.d. = 0.02 cents s^−1^), and for the heavy-tailed timing environment it was 1.62 cents s^−1^ (s.d. = 0.02 cents s^−1^). This difference was statistically significant (*t*_47_ = –2.46, *p* = 0.018), demonstrating that, on average, participants achieved a higher average reward rate in the heavy-tailed timing environment.

### (b) Differences between drink condition

Having identified that participants altered their temporal persistence for each timing environment, we next assessed whether caloric reward had an effect on temporal persistence. To do this, we used a full factorial shared lognormal frailty model, with both treatment condition and timing environment, and their interaction, as regressors. We used data only from the blocks in which participants consumed liquid.

We re-established the significant effect of the heavy-tailed timing environment (*β* = 1.45, s.e. = 0.05, *z* = 27.68, *p* < 0.001), which had a hazard ratio of 4.25 (CI = 3.84–4.71). We also observed a significant main effect of the caloric reward (*β* = 0.3, s.e. = 0.06, *z* = 4.97, *p* < 0.001), with a hazard ratio of 1.35 (CI = 1.2–1.52). Further to this, we also found a significant interaction effect between the heavy-tailed timing environment and the caloric reward (*β* = −0.5, s.e. = 0.07, *z* = −7.58, *p* < 0.001), with a hazard ratio of 0.60 (CI = 0.53–0.69).

**Figure 3.**
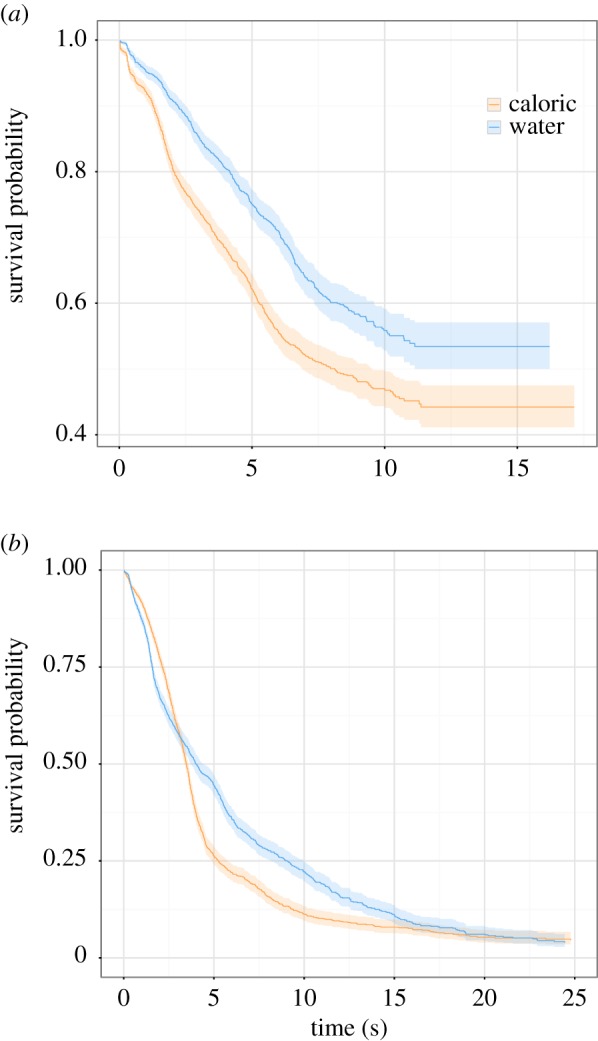
(*a*) Survivor functions for treatment conditions within the uniform timing environment. The probability of waiting was lower in the caloric treatment condition relative to the water treatment condition. (*b*) Survivor functions for treatment conditions within the heavy-tailed timing environment. The probability of waiting was generally lower in the caloric treatment condition relative to the water treatment condition. Note that all functions and shaded confidence intervals are estimated from pooled data.

To compute hazard ratios for the effect of treatment condition within each timing environment, we created two ‘simple effects’ models treating the uniform and heavy-tailed timing environments separately. Within the uniform timing environment, the model re-established a significant effect of the caloric reward (*β* = 0.23, s.e. = 0.08, *z* = 3.04, *p* = 0.002). The hazard ratio of the caloric reward group was 1.26 (CI = 1.08–1.46), indicating that, relative to water, the probability of quitting was more likely after consuming caloric reward. Survivor functions estimated using pooled data for each treatment condition in the uniform timing environment are shown in figure 3*a*.

We also found a significant effect of the caloric reward within the heavy-tailed timing environment (*β* = 0.11, s.e. = 0.05, *z* = 2.15, *p* = 0.032), with a hazard ratio of 1.12 (CI = 1.01–1.23), indicating that relative to water, the probability of quitting was more likely after consuming caloric reward. Survivor functions estimated using pooled data for each treatment condition in the heavy-tailed timing environment are shown in figure 3*b*.

The effects in all three of these models remained significant after correction for multiple comparisons. The observed pattern of results suggested that overall, caloric reward increased quitting probabilities, but to a smaller degree within the heavy-tailed timing environment.

We then ran an analysis to identify whether the caloric treatment condition affected experienced reward rate as a result of the increase in quitting probability, compared to the control condition. To do this, we used only data from blocks in which participants consumed liquid, and treated each timing environment separately.

The mean experienced reward rates in the uniform timing environment were 1.51 (s.d. = 0.03) and 1.53 (s.d. = 0.03) cents per second, for the caloric and control conditions, respectively. This difference was not significant (*t*_45.44_ = –0.21, *p* = 0.836). In the heavy-tailed timing environment, the mean experienced reward rates were 1.78 (s.d. = 0.02) and 1.64 (s.d. = 0.02) cents per second, for the caloric and control conditions, respectively. This difference was significant (*t*_45.83_ = 2.16, *p* = 0.036). Overall, this suggests that the increase in quitting probability due to the caloric treatment affected earnings only in the heavy-tailed timing environment.

### (c) Effect of drink consumption versus no consumption

We further subdivided the data to assess how liquid consumption and liquid type affected the probability of quitting in each timing environment. For each timing environment, and for each liquid type, we estimated a shared lognormal frailty model to identify whether liquid consumption altered temporal persistence relative to non-consumption, within each group.

Within the uniform timing environment, consumption of water did not significantly affect the probability of quitting (*β* = 0.003, s.e. = 0.05, *z* = 0.05, *p* = 0.959). However, consumption of caloric reward did significantly affect the probability of quitting (*β* = 0.28, s.e. = 0.05, *z* = 6.21, *p* < 0.001). This was equivalent to a hazard ratio of 1.32 (CI = 1.21–1.44), and suggests that the consumption of caloric reward decreased temporal persistence, relative to blocks in which the same participants did not consume liquid.

Within the heavy-tailed timing environment, the consumption of water had a significant effect on the probability of quitting (*β* = –0.14, s.e. = 0.03, *z* = –3.96, *p* < 0.001), equivalent to a hazard ratio of 0.87 (CI = 0.81–0.93). This suggested that water consumption increased temporal persistence, relative to blocks in which the same participants did not consume liquid. The consumption of caloric reward also had a significant effect on quitting probability (*β* = 0.13, s.e. = 0.03, *z* = 3.89, *p* < 0.001), equivalent to a hazard ratio of 1.14 (CI = 1.07–1.22).

With the exception of water consumption in the uniform timing environment, the effects of drink consumption in both timing environments were significant after correction for multiple comparisons. Overall, these results suggest that, relative to blocks in which there was no liquid consumption, there was a systematic effect of the consumption of caloric reward across both timing environments, such that temporal persistence was decreased. They also suggest that water consumption significantly increased temporal persistence, but only within the heavy-tailed timing environment.

## 4. Discussion

In this study, we assessed whether individuals changed their persistence in waiting for monetary reward as a result of differences in timing environment (average experienced reward rate) and differences in energy balance. In line with our predictions, we found that temporal persistence was lower in the presence of a higher average reward rate and when participants consumed a caloric drink. Decision-makers should make quitting decisions when the opportunity cost of waiting exceeds a certain threshold [7], or when they are averse to the risk inherent in delay [1,2]. Thus, we interpret these relatively expedited quitting times as a consequence of increased subjective opportunity costs [10], and increased risk-aversion (in line with the energy budget rule; [22]), respectively.

The neurocomputational model of Niv *et al.* [10] predicts that the latency of actions should decrease when average reward rate is high, in order to offset the relatively high opportunity cost of time [10]. Our results support this prediction by indicating lower temporal persistence when in an environment with a higher average reward rate. This is consistent with the results of a previous study using the same paradigm and similar timing environments [3], as well as previous studies employing different paradigms that have manipulated reward rates to show effects on vigour, measured by reaction times ([11,13], and see [29]), and the force of responding [12]. Thus, our findings further support the notion that reward rate and opportunity cost (possibly encoded by tonic dopamine) can affect timing over a broad range of tasks and timescales. This appears to apply to motor control (i.e. reaction times, [19]), perception (i.e. time perception, [15,16,19]), as well as decision making (delay discounting, [17]; and now temporal persistence). In sum, these findings point to a general, reward-sensitive mechanism that calibrates many time-related functions.

One alternative interpretation of this result is that participants tailored their persistence by estimating the exact temporal distribution of rewards, rather than the average reward rate. However, previous work has suggested that humans are more likely to use heuristic approaches—such as average experienced reward rate—to determine the latency of actions [10,30]. This question could be further investigated by comparing temporal persistence under timing conditions that have identical reward rates, but distinct statistical distributions. We also note that while the apparent difference in average experienced reward rate between the timing environments was small, it has been argued that the matching of behaviour to reward rates is innate [31], and thus participants may not have needed to be explicitly aware of differences between the reward rates in each timing environment to alter their behaviour.

We also found that relative to those who consumed water, participants who consumed a sweet, caloric drink had decreased temporal persistence. Given that delays carry implicit uncertainty [1,2], the energy budget rule predicts that temporal persistence should be lower when energy balance is positive [22]. Our results constitute the first empirical demonstration of this in humans. A possible alternative explanation for this result is that caloric rewards increased task performance, as they have been shown to do in other cognitive domains [32]. However, optimal task performance in the uniform timing environment required prolonged persistence, which directly conflicts with the observed effect of caloric rewards in this condition.

In our experiment, participants who consumed water did not systematically alter their temporal persistence, which suggests that this effect was due specifically to the flavour or energy content in the caloric reward, rather than primary rewards in general. As we combined both maltodextrin and aspartame in the caloric treatment condition, we are unable to disassociate the alimentary and hedonic aspects of the liquid, i.e. whether the caloric content of the liquid is sufficient to decrease temporal persistence, or whether a sweet flavour must also be present. However, previous studies have shown that calorie-rich nutrients affect reward pathways independently of palatability [33–35], and tasteless carbohydrates can affect aspects of behaviour, such as exercise performance [36,37] and self-control [32], whereas artificially sweetened solutions do not. Thus, while we are unable to attribute the effect to caloric reward with certainty, we find it more likely that caloric content—rather than sweetness—affected behaviour. The possibility that either caloric content or flavour is responsible for the effect on temporal persistence could be explored in a future study.

We also note that for the heavy-tailed timing environment, participants who consumed water (who had relatively negative energy balance) abandoned waiting later than was optimal. Thus, for our experimental design, the energy budget rule for temporal persistence appeared to be maladaptive. One possible explanation for this is that our timing environments are not perfectly representative of ecological reward timing distributions. Future studies could address this by assessing task performance in a wider range of timing distributions.

Caloric rewards have previously been proposed to enhance behaviour in the cognitive domain of self-control [38], which would suggest that caloric reward might have increased, rather than decreased temporal persistence. Indeed, a number of studies have reported that calories and other satiety factors have such an effect. For instance, glucose consumption has been shown to enhance patience in intertemporal choice (compared with artificial sweeteners [39]; or fructose [40]). Similarly, it has been shown that the ‘hunger hormone’ ghrelin decreases patience [41], suggesting that a low energy budget increases subjective temporal opportunity costs. In addition, there is evidence that the consumption of fruit juice rewards can cause underestimations of time [42], which implies a ‘slower’ subjective pacemaker, and therefore a lower subjective temporal opportunity cost. These studies recapitulate the idea that opportunity costs ought to decrease as organisms become satiated, as there is a less urgent requirement for nutrition. Thus, an alternative prediction for our experiment would have been that physiological rewards (such as the sweet, caloric solution used in our experiment) would decrease temporal opportunity costs and increase temporal persistence. However, our results do not support this. Instead, we speculate that the consumption of calories led to elevated tonic dopamine levels in reward-related midbrain areas, rather than an inhibition of dopaminergic activity, as has previously been suggested [43], although we note that we do not have direct evidence for this. However, an increase in tonic levels of dopamine in response to calorie consumption is consistent with human imaging studies [35,44,45]. It would also be consistent with the time perception literature, where increasing dopamine levels leads to earlier time estimations [15,16]. Furthermore, similar effects have been reported in other reward-based decision making tasks, where increases in dopamine levels have been shown to increase impulsive choices [17] and actions [46].

Our results show that heightened reward rate and increased energy balance appear to affect temporal persistence in a similar way. One apparent question is whether these effects are facilitated by a common mechanism. Previous research has suggested that the value of money is a modern derivative of the desire for primary rewards [47], and that the predictions of the energy budget rule are met when average monetary reward rate is used as a proxy for metabolic energy balance [48]. Likewise, money and juice reinforcers cause overlapping neural responses [49]. It is therefore possible that both high monetary reward rates and the consumption of calories affect behaviour via a common psychophysiological mechanism, i.e. they both signal the general availability of reward. This should result in an increase in the perceived opportunity cost of time and increased motivational drive, whether the source is external [10] or internal [50]. However, if such a common mechanism was limited in scalability, increasing opportunity cost would not have an unlimited effect on behaviour. This may account for the smaller effect of positive energy balance in the heavy-tailed environment, where these two factors were combined. Given the hypothesis of a common mechanism, and the fact that energy balance non-specifically affected temporal persistence towards monetary goals, it would be of interest for future work to determine whether monetary reward can also affect temporal persistence toward food-related goals.

Impulsivity is a broad, but heavily studied construct in psychology, economics and psychiatry [51], and relates to a wide range of psychiatric disorders [52]. An individual's temporal persistence—as measured by this paradigm—may constitute a useful measure of impulsivity, as a lack of temporal persistence would imply an inability to delay gratification, and a higher likelihood reneging on a long-term goal (i.e. preference reversals [3]). The dominant behavioural model of impulsivity is intertemporal choice, which has been shown to be a relatively stable and heritable trait [52]. However, when used in laboratory experiments, intertemporal choice rarely engenders actual temporal opportunity costs, as individuals are not required to forgo alternatives while waiting for delayed rewards. Similarly, the delays to reward receipt are usually well specified, and the one-shot nature of the task does not allow for preference reversals. This highlights a distinction between impulsive choice and impulsive action, which capture different aspects of impulsive behaviour [53]. The temporal persistence task used in this experiment may involve a combination of these constructs: the trade-off between short- and long-term rewards commonly associated with impulsive choice, as well as the capacity to inhibit prepotent responses that is relevant to impulsive action. Given that our primary measures are responses to concurrent opportunity costs and time pressure, we consider our task to be perhaps more relevant to action impulsivity. Importantly, while the task only requires passive waiting to acquire larger rewards (rather than, for instance, physical effort), the uncertainty of the delays and possibility for preference reversals may be more representative of real decision scenarios. This may explain the discrepancy between the results of our experiment, and those that have used intertemporal choice to show that positive energy balance can increase patience [39]. A further question raised by our results is, therefore, whether the effect of increased energy balance on temporal persistence applies to impulsive behaviour more generally. This possibility may provide a promising avenue for further research.

In conclusion, in addition to demonstrating that individuals calibrate their temporal persistence depending on average experienced reward rates, we also demonstrated a clear effect of caloric reward on temporal persistence. Previous work has shown an effect of average experienced reward rates on reaction times and the force of responses [11–13], and we extend this effect to temporal persistence. Previous studies have also identified that humans become more risk-averse when satiated [23,24], but our study is the first to demonstrate that energy balance affects temporal persistence. This contributes to growing evidence that physiological rewards play a crucial role in modulating cognition and decision-making [21,39–41].

## Data accessibility

All data are available at the Dryad Digital Repository: http://dx.doi.org/10.5061/dryad.1k79k [54].
